# Supplementary material for: Resolving the full spectrum of human genome variation using Linked-Reads
Source: Genome Res. 2019 Apr;29(4):635–45. doi: 10.1101/gr.234443.118 (PMC6442396; doi:10.1101/gr.234443.118)
Supplement: Supplemental Material [file supp_gr.234443.118_Supplemental_longranger-2.2.zip › longranger-2.2.tar.bz2!/lr2.2-public/lib/stan/cmdstan/stan/doc/api/html/namespacestan_1_1mcmc.html]

Stan: stan::mcmc Namespace Reference


|  |  |
| --- | --- |
|  | Stan  2.5.0  probability, sampling & optimization |


- Main Page
- Related Pages
- Namespaces
- Classes
- Files

- Namespace List
- Namespace Members

All Classes Namespaces Files Functions Variables Typedefs Enumerations Enumerator Friends Macros Pages

- stan
- mcmc

Classes

stan::mcmc Namespace Reference

Markov chain Monte Carlo samplers.
More...

|  |  |
| --- | --- |
| Classes | |
| class | base\_adaptation |
|  | |
| class | base\_adapter |
|  | |
| class | base\_mcmc |
|  | |
| class | chains |
|  | An `mcmc::chains` object stores parameter names and dimensionalities along with samples from multiple chains. More... |
|  | |
| class | covar\_adaptation |
|  | |
| class | fixed\_param\_sampler |
|  | |
| class | base\_hmc |
|  | |
| class | base\_hamiltonian |
|  | |
| class | dense\_e\_metric |
|  | |
| class | dense\_e\_point |
|  | |
| class | diag\_e\_metric |
|  | |
| class | diag\_e\_point |
|  | |
| class | ps\_point |
|  | |
| class | unit\_e\_metric |
|  | |
| class | unit\_e\_point |
|  | |
| class | base\_integrator |
|  | |
| class | base\_leapfrog |
|  | |
| class | expl\_leapfrog |
|  | |
| class | adapt\_dense\_e\_nuts |
|  | |
| class | adapt\_diag\_e\_nuts |
|  | |
| class | adapt\_unit\_e\_nuts |
|  | |
| struct | nuts\_util |
|  | |
| class | base\_nuts |
|  | |
| class | dense\_e\_nuts |
|  | |
| class | diag\_e\_nuts |
|  | |
| class | unit\_e\_nuts |
|  | |
| class | adapt\_dense\_e\_static\_hmc |
|  | |
| class | adapt\_diag\_e\_static\_hmc |
|  | |
| class | adapt\_unit\_e\_static\_hmc |
|  | |
| class | base\_static\_hmc |
|  | |
| class | dense\_e\_static\_hmc |
|  | |
| class | diag\_e\_static\_hmc |
|  | |
| class | unit\_e\_static\_hmc |
|  | |
| class | sample |
|  | |
| class | stepsize\_adaptation |
|  | |
| class | stepsize\_adapter |
|  | |
| class | stepsize\_covar\_adapter |
|  | |
| class | stepsize\_var\_adapter |
|  | |
| class | var\_adaptation |
|  | |
| class | windowed\_adaptation |
|  | |

## Detailed Description

Markov chain Monte Carlo samplers.


---

[ Stan Home Page ]

*© 2011–2014,
Stan Development Team.*
